# Major cardiovascular disease (CVD) risk factors in midlife and extreme longevity

**DOI:** 10.1007/s40520-019-01364-7

**Published:** 2019-10-14

**Authors:** Annele Urtamo, Satu K. Jyväkorpi, Hannu Kautiainen, Kaisu H. Pitkälä, Timo E. Strandberg

**Affiliations:** 1 Department of General Practice and Primary Health Care, Helsinki University Central Hospital, Unit of Primary Health Care, University of Helsinki, Helsinki, Finland; 2 Clinicum, and Helsinki University Hospital, University of Helsinki, Helsinki, Finland; 3 Center for Life Course Health Research, University of Oulu, Oulu, Finland

**Keywords:** Longevity, Successful ageing, Nonagenarians, Life-course, Cardiovascular risk factors

## Abstract

**Background:**

The studies on the association of various midlife risk factors with reaching 90 years or more are scarce. We studied this association in a socioeconomically homogenous cohort of businessmen.

**Methods:**

The study consists of men (*n* = 970) from the Helsinki Businessmen Study cohort (born 1919–1928). Five major cardiovascular disease (CVD) risk factors (smoking, BMI, blood pressure, serum lipids, fasting glucose), consumption of alcohol and coffee, self-rated health and self-rated fitness, were assessed in 1974, at an average age of 50 years. The number of major risk factors was tested as a risk burden. The Charlson Comorbidity Index and the RAND-36 (SF-36) Physical and Mental health summary scores were calculated from surveys in year 2000, at age of 73 years. Mortality dates were retrieved through 31 March 2018 from the Population Information System of Finland.

**Results:**

244 men survived to the age of 90 representing 25.2% of the study cohort. The survivors had less risk factor burden in midlife, and less morbidity and higher physical health summary score in 2000. Of those with five major risk factors only 7% survived up to 90 years, whereas 51% of those without any risk factors reached that age. Single risk factors reducing odds of reaching 90 years were smoking (odds ratio [OR] 0.48, 95% confidence interval [CI] 0.34–0.67), glucose (0.66, 0.49–0.88), BMI (0.63, 0.46–0.86), and cholesterol (0.71, 0.53–0.96).

**Conclusion:**

Lack of five major CVD risk factors in midlife strongly increased odds of reaching 90 years of age and also predicted factors related to successful ageing in late life.

## Introduction

Life expectancy has remarkably increased in Europe in the past decades [1]. Increasing numbers of people reach exceptional longevity, 90 years or more [1]. For example, in Finland the probability of a 50-year old man of reaching the age of 90 has increased from 12 to 20% in 44 years [2].

Earlier studies have revealed the multitude of factors associated with longevity [3–11]: genetic factors may explain approximately 25% of the variation of lifespan, while healthy lifestyle habits in midlife also play an important role [12].

For exceptional longevity, Yates et al. [13] studied the association of factors in age of 70 with survival till 90, and they revealed that the probability of reaching 90 years of age was 54% for those men who did not have any risk factors, and 4% for those who had five risk factors (smoking, diabetes, obesity, hypertension, sedentary lifestyle) at mean age of 72. In addition, a Swedish prospective study [14] among a heterogeneous cohort showed that non-smoking, socioeconomic status and general good health in midlife were strong predictors of survival to the age of 90.

To our knowledge, there are scarce prospective studies in homogeneous cohorts with very long follow-ups, especially exploring how combination of midlife major risk factors determines reaching 90 years of age. We investigated these midlife factors in a 44-year follow-up study of the Helsinki Businessmen Study cohort in which socioeconomic effects on longevity are inherently adjusted for. To reflect the quality of longer life, we also compared some factors related to “successful” ageing at the age of 73 years between those who did or did not reach 90 years of age.

## Design and methods

### Study population

HBS is a prospective cohort study of initially healthy Finnish businessmen and executives born between 1919 and 1934 (original *n* = 3490). Details of the study cohort have been described earlier [15]. The present study included those men who had the possibility to reach 90 years of age by March 2018 (born between 1919 and 1928) and had data of all major risk factors available in 1974 (*n* = 970). This sub-group has been followed-up for 44 years with mailed questionnaires, and clinical and laboratory assessments. The follow-up was approved by the Ethics Committee of the Helsinki University Hospital, Department of Medicine, and all participants had provided informed consents.

### Baseline measurements

Midlife factors were assessed in 1974, at an average age of 50 years. Clinical and laboratory examinations included body mass index (BMI, kg/m^2^), systolic and diastolic blood pressure (mmHg), total serum cholesterol (mmol/L), triglycerides (mmol/L) and 1-h post-load glucose (mmol/L). In addition, the status of smoking and consumption of coffee (cups/day) and alcohol (g/week) were asked in a questionnaire. Major cardiovascular risk factors included smoking status (yes/no), systolic blood pressure (≥ 144 mmHg), total cholesterol (> 6.2), body mass index (> 25) and fasting glucose (≥ 4.7). Median was used as a cut-point for risk in continuous variables. The number of major risk factors was tested as a risk burden.

Self-rated health (SRH) and self-rated fitness (SRF) were assessed by asking “What do you think about your present state of health/fitness; is it “very good”, “fairly good”, “average”, “fairly poor” or “very poor”. The status as “very good” and “fairly good” were combined in analyses of SRH and SRF.

In 2000, men were sent a mailed questionnaire which included the survey of diseases and the validated Finnish version of the RAND-36 Health Survey [16]. The Charlson Comorbidity Index (CCI) was used as a measure of burden of disease [17]. The Physical and Mental health summary scores of the RAND-36 Health Survey were calculated and used in the analyses [18].

### Mortality

**Fig. 1:**
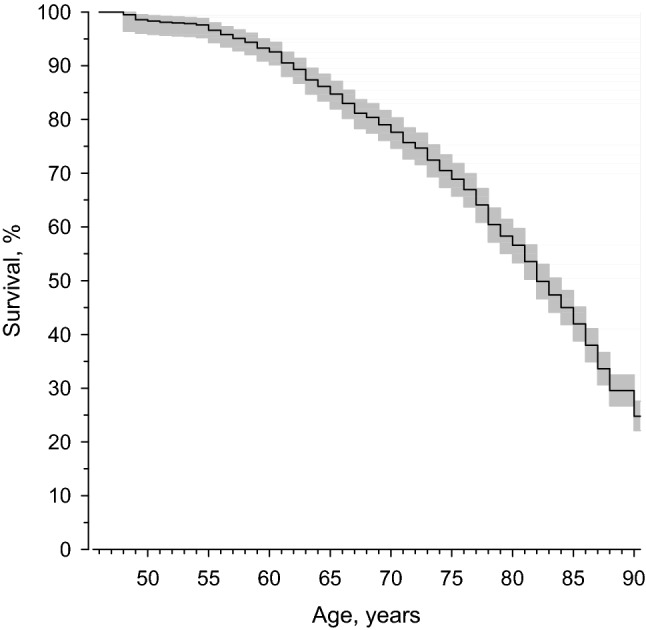
Survival to age of 90 among HBS sub-cohort, *n* = 970

The dates of death were drawn from the national Population Information System in March 2018. Between years 1974 and 2018, 830 men of this sub-cohort had died. Of the deceased persons, 104 had reached 90 years of age whereas 726 had died before the age of 90 (Fig. 1).

### Statistical analyses

The descriptive statistics are presented as means with SDs or as counts with percentages. Statistical comparisons between the groups were made using the *t* test or Chi square test. In the case of violation of the assumptions (e.g., non-normality), a bootstrap-type test was used. Kaplan–Meier method with age is the time scale used to estimate the cumulative survival. To determine characteristics associated with reaching 90 years of age, multivariate logistic regression analysis was applied. The normality of the variables was tested using the Shapiro–Wilk *W* test. Stata 15.1 (StataCorp LP, College Station, TX, USA) was used for the analysis.

## Results

The baseline characteristics of the study cohort in 1974 are shown in Table 1. The mean age was 50 years (range 46–55 years). 25.2% (*n* = 244) men of the cohort had reached 90 years or more by March 2018. Survivors to the age of 90 had less likely smoking history, and lower BMI than non-survivors. They had also lower levels of both systolic and diastolic blood pressure, fasting and 1-h glucose and serum lipids. They also consumed less alcohol. There were slight differences in midlife SRH and SRF between survivors to the age of 90 and non-survivors, but those were not significant.

**Table 1:** Characteristics of study population (*n* = 970)

Variables in 1974	All *n* = 970	Reached 90 *n* = 244	Not reached 90 *n* = 726	*p* value for difference between groups
Age, years	50 (2.7)	50 (2.7)	50 (2.7)	0.90
Body mass index (kg/m^2^)	26.1 (2.8)	25.4 (2.6)	26.3 (2.9)	< 0.001
Smoking *n* (%)	341 (35)	58 (24)	283 (40)	< 0.001
Blood pressure (mmHg)				
Systolic	146 (20)	143 (20)	147 (20)	0.023
Diastolic	94 (12)	92 (12)	94 (12)	0.004
Serum lipids (mmol/L)				
Cholesterol	6.37 (1.10)	6.13 (1.03)	6.47 (1.11)	< 0.001
Triglycerides	1.69 (0.89)	1.55 (0.75)	1.74 (0.93)	0.004
1-h post-load glucose (mmol/L)	7.63 (2.30)	7.15 (2.12)	7.80 (2.33)	< 0.001
Alcohol (g/week)	161 (169)	136 (133)	170 (178)	0.008
Coffee (cups/day)	4.1 (2.5)	3.8 (2.4)	4.1 (2.5)	0.13
Self-rated health (SRH), rated as good, *n* (%)	461 (49)	120 (52)	341 (48)	0.69
Self-rated fitness (SRF), rated as good, *n* (%)	317 (34)	87 (38)	230 (32)	0.069
Variables in 2000				
Physical Health summary	44 (9) [515]	46 (8) [208]	43 (10) [307]	< 0.001
Mental Health summary	53 (10) [515]	54 (10) [208]	53 (10) [307]	0.39
Charlson	1.5 (1.4) [548]	1.1 (1.2) [215]	1.8 (1.5) [333]	< 0.001

Continuous variables are mean (SD)

In 2000, the Charlson comorbidity index was significantly lower for men who reached 90 years of age. In addition, the Physical health summary score in RAND-36 was significantly higher for those who survived till age of 90, but differences in the Mental health summary score were not significantly different.

**Fig. 2:**
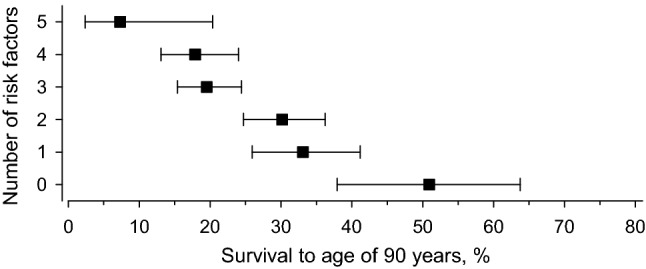
Association of the amount of cardiovascular risk factors with survival to age of 90

Of those who had none of the major risk factors, 51% reached 90 years of age. With one major risk factor, the percentage was 33%, with two risk factors 30%, with three risk factors 20%, and with four risk factors 18%. Whereas of those with five major risk factors only 7% survived until 90. There was a linear relationship between the number of risk factors and reaching up to 90 years of age (Fig. 2).

In logistic regression analysis, significant risk factors were smoking (OR 0.48, 95% CI 0.34–0.67), glucose (OR 0.66, 95% CI 0.49–0.88), BMI (OR 0.63, 95% CI 0.46–0.86) and cholesterol (OR 0.71, 95% CI 0.53–0.96) whereas systolic blood pressure did not reach significance. Table 2.

**Table 2:** Odds for reaching 90 among the HBS cohort

Variable (adjusted for age)	Multivariate^a^	
	OR (95% CI)	*p* value
Smoking	0.48 (0.34–0.67)	**< 0.001**
Systolic blood pressure	0.88 (0.65–1.19)	0.412
Body mass index	0.63 (0.46–0.86)	**0.003**
1-h post-load glucose	0.66 (0.49–0.88)	**0.006**
Total cholesterol	0.71 (0.53–0.96)	**0.027**
Alcohol	0.83 (0.60–1.14)	0.24
Self-rated health (SRH)	1.00 (0.69–1.44)	0.99
Self-rated fitness (SRF)	1.05 (0.72–1.55)	0.80

Bold values indicate the significant values (*p* < .05)

^a^ represents forward stepwise selection. Only those variables which entered the model are shown

## Discussion

Our findings revealed that the burden of major cardiovascular risk factors is a linear and strong determinant of mortality and this burden efficiently prevents reaching 90 years of age. Of those having none of these major risk factors 51% survived until 90 years of age whereas among those with five risk factors only 7% reached that age. Smoking, higher BMI, and higher levels of glucose and cholesterol were significant predictors for mortality in logistic regression analysis and prevented survival to the age of 90. The survivors had significantly less chronic diseases at the mean age of 73 years, and their physical health summary score was also significantly higher than among non-survivors suggesting that the longer life span was not necessarily of poor quality.

Previous studies have reported that absence of various risk factors in midlife is associated with longer life expectancy [11, 14, 19–21]. Similar to our study, the burden of risk factors has been studied in the Framingham Heart Study cohort, but the study population was socioeconomically more diverse, and the educational level was also found to be associated with the survival to exceptional longevity [21]. Also, Yates et al. [13] have studied the cumulative effect of cardiovascular risk factors, and the results were similar to ours. However, the factors were measured at age 20 years older than in our cohort, and therefore the follow-up time was much shorter, 18 years. Yates et al. [13] found that the SRH was significantly higher at mean age of 72 among those who survived to the age of 90. However, there was no difference in SRH between those who did or did not reach 90 years in our study with a 44-year follow-up. This may be due to the fact that SRH has better prognostic value in shorter follow-ups when an individual has more accurate information about one’s condition [22].

The study of Wilhelmsen et al. [14] showed that high socioeconomic status and good functional capacity were associated with the survival to the age of 90 years [14]. In our study, the physical health summary score at mean age of 73 was higher among those businessmen who reached 90 years of age. Also, at the mean age of 73 those who did not survive up to that age had more disability and morbidity than survivors.

In midlife, the future survivors to 90 years of age had a significantly lower average systolic and diastolic blood pressure than non-survivors. However, in contrast to previous studies [13, 14, 20, 21], blood pressure was not an independent predictor of survival to the age of 90. This can be explained by correlation of blood pressure with BMI and smoking. Furthermore, antihypertensive treatments during follow-up may also have diluted potential differences.

The proportion of people who reach 90 years of age has increased over the past century [3]. During past decades the life expectancy has increased especially for the people who already have entered old age [3]. The concept of successful ageing has been generally associated with longevity, highlighting the importance of a very long, healthy and active life [3, 4]. The important aspect of this paradigm is ‘‘the compression of morbidity’’ explaining that disabilities and illnesses can be packed to a minimum period in later years and thus expand the healthy life span [3, 4]. Our results also support this theory. Prevention and advanced treatment of chronic diseases enable an individual to maintain functionality and age “successfully” [23–32]. Cardiovascular risk factors are well known, and their treatments have developed during past decades [33], although intensity of treatment varies between various risk factors despite effective treatments for, e.g., hypertension and dyslipidemia. The obesity epidemic, in turn, continues to lack effective treatments [33]. For aiming exceptional longevity, our results nevertheless highlight the cumulative effects of common risk factors, which should be taken account in practice and in health promotion interventions.

Longitudinal data on a combination of risk factors related to survival beyond 90 years have been limited. The strength of our study is the very long, 44-year follow-up of initially healthy men. Our study sample is also very homogeneous representing men from the upper social class which reduces confounding by socioeconomic factors. This is obviously also a limitation for the generalizability of the results. In addition, the cumulative CVD risk factors were only assessed at an average age of 50, and the quality of life and morbidity were only reported at an average age of 73 years. Furthermore, the analyses do not capture the variables of social context.

### Implications

Our results show that the burden of midlife risk factors is associated with survival to advanced ages, and suggest that having a healthy lifestyle in midlife, especially avoiding smoking and obesity, may improve the probability of reaching exceptional longevity, 90 years or more. Therefore, the study gives important incentives for individuals and practitioners who wish to promote longevity.
